# Production of HMF-derivatives from wine residues using *Saccharomyces cerevisiae* as whole-cell biocatalyst

**DOI:** 10.1186/s40643-025-00840-5

**Published:** 2025-01-31

**Authors:** Joana T. Cunha, Aloia Romaní, Lucília Domingues

**Affiliations:** 1 CEB - Centre of Biological Engineering, University of Minho, Campus Gualtar, Braga, 4710-057 Portugal; 2 LABBELS –Associate Laboratory, Braga/Guimarães, Portugal; 3 Departamento de Enxeñaría Química, Facultade de Ciencias, Universidade de Vigo, Ourense, 32004 Spain; 4 Instituto de Agroecoloxía e Alimentación (IAA), Universidade de Vigo, Campus Auga, Ourense, 32004 Spain

**Keywords:** HMF, FDCA, Biocatalysis, *Saccharomyces cerevisiae*, Microwave heating technology, Wine byproducts, Circular economy

## Abstract

**Background:**

There is an urgent need to develop bioprocesses independent of fossil resources to address resource depletion and mitigate environmental harm. Transitioning to a bio-based economy requires prioritizing chemical production processes that utilize renewable resources, ensuring sustainability and environmental responsibility. 5-Hydroxymethylfurfural (HMF) and its derivatives are promising building blocks, ranked among the top 12 bio-based molecules derived from biomass. This study investigates the potential of wine residues as substrates for HMF production and explores the yeast *Saccharomyces cerevisiae*, a robust industrial microbial cell factory, as a whole-cell biocatalyst for converting HMF into high-value compounds, offering an alternative to chemical synthesis.

**Findings:**

Several *S. cerevisiae* strains were compared for their ability to convert HMF, demonstrating varying capacities for oxidation or reduction. For the first time, HMF derivatives with potential industrial applications were produced using an HMF-rich hydrolysate obtained from sustainable processing of wine-growing waste, such as grape pomace and must surplus. The selected yeast strain was engineered to express the oxidoreductase enzyme of HMF/Furfural from *Cupriavidua basilensis* strain HMF14, resulting in a 15-fold increase in the accumulation of oxidized derivatives such as 2,5-furandicarboxylic acid (FDCA).

**Conclusions:**

These findings highlight the potential of leveraging wine residues and engineered *S. cerevisiae* strains to develop sustainable bioprocesses for producing valuable HMF derivatives, thereby contributing to the advancement of bio-based chemical production.

**Graphical Abstract:**

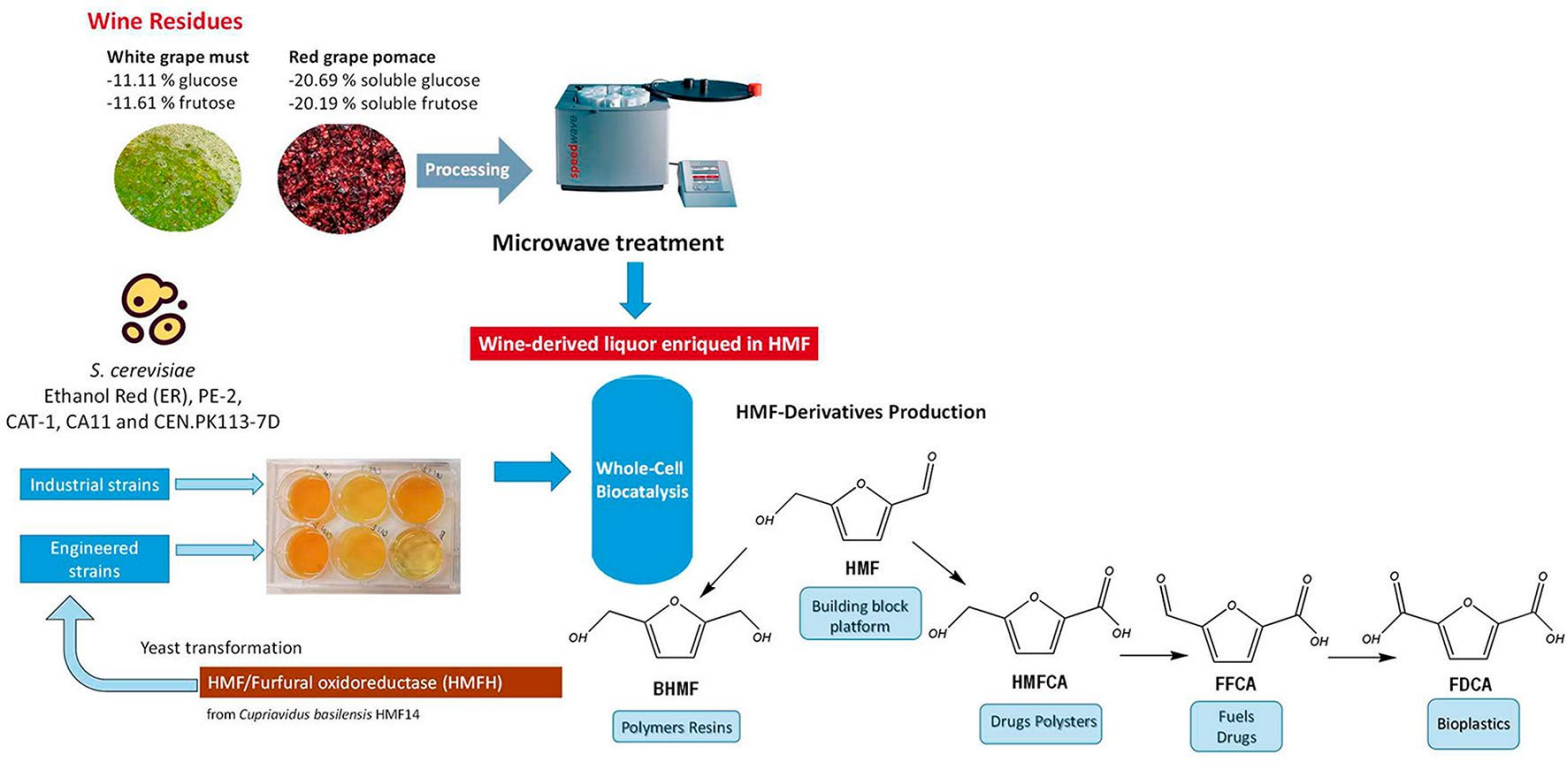

**Supplementary Information:**

The online version contains supplementary material available at 10.1186/s40643-025-00840-5.

## Introduction

Developing biobased processes is essential for reducing dependence on fossil resources, lowering greenhouse gas emissions, and mitigating pollution. Transitioning to renewable feedstocks, offers a promising solution, enabling the production of high-value chemicals in a more sustainable and environmentally friendly manner. In this context, 5-hydroxymethylfurfural (HMF) is recognised as a versatile platform of renewable chemicals obtained from the dehydration of hexoses, which are present in a variety of renewable resources (Cunha et al. 2022). The wine industry generates substantial quantities of waste streams, including excess grape must and grape pomace, which present environmental challenge if not properly managed (Jesus et al. 2022; Baptista et al. 2023). These residues, rich in hexoses, are well-suited for conversion into HMF, contributing to their sustainable valorization within a biorefinery framework (Kalli et al. 2018). For the catalytic production of HMF from winery wastes, emerging heating technologies such as microwaves have been increasingly employed due to their advantages, including cost-effective pretreatment and reduced reaction times (Pérez-Pérez et al., 2023). These benefits help minimize side reactions in aqueous media.

**Fig. 1:**
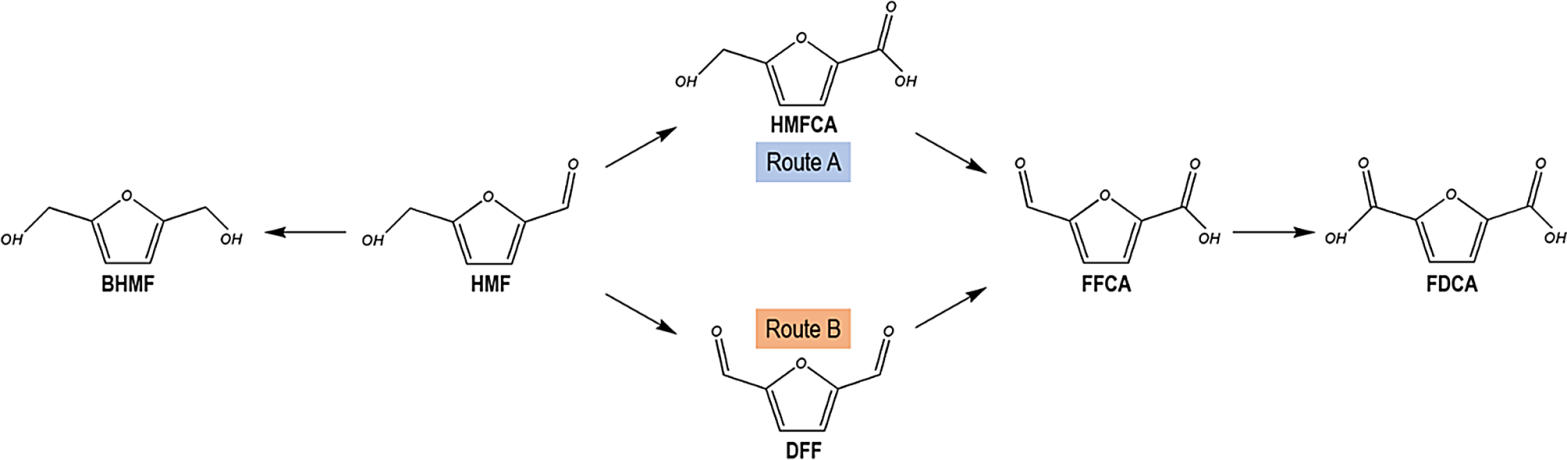
Routes for the reduction or oxidation of HMF and main applications of HMF-derivatives. BHMF: 2,5-bis(hydroxymethyl)furan. HMF: 5-hydroxymethylfurfural. HMFCA: 5‐hydroxymethyl‐furan‐2‐carboxylic acid. DFF: 2,5-diformylfuran. FFCA: 5‐formyl‐2‐furancarboxylic acid. FDCA: 2,5‐furandicarboxylic acid

As a bio-based chemical platform, HMF is a precursor for the synthesis of other chemicals via oxidation or reduction reactions (Cunha et al. 2022). The oxidation process follows two pathways, as shown in Fig. 1. HMF derivatives have a wide range of industrial applications, including the manufacture of polyurethane foams (2,5-bis(hydroxymethyl)furan-BHMF). On the other hand, oxidative HMF-derived compounds have diverse industrial uses. For example, 5-hydroxymethyl‐furan‐2‐carboxylic acid (HMFCA) is used in polyester production and as an antitumor agent, while 2,5-diformylfuran (DFF) serves as a precursor for synthesizing fungicides, novel polymeric materials, and pharmaceuticals. Additionally, 5‐formyl 2‐furancarboxylic acid (FFCA) is employed in the production of resins and surfactants. Notably, 2,5-furandicarboxylic acid (FDCA) has the potential to replace isophthalic, adipic, and terephthalic acids in the manufacture of polyamides, polyesters and polyurethanes (Saikia et al. 2021). In fact, FDCA stands out as a versatile starting compound and one of the most important biomass-derived chemicals (Bozell and Petersen 2010). FDCA is primarily used to produce polyethylene furanoate, a sustainable substitute to petrochemical-derived polyethylene terephthalate (PET) plastic. Due to its vast potential, FDCA has been labeled a ‘sleeping giant’ in the field of renewable chemicals (Rajesh et al. 2020). Therefore, HMF-based products have similarly garnered growing attention for their role in advancing sustainable chemical innovations. Nonetheless, the synthesis of these compounds has predominantly relied on chemical processes characterized by high-cost catalysts, harsh reaction conditions, and limited specificity (Hu et al. 2018). In recent years, biological catalysts for HMF have emerged as a greener and more promising alternative to chemical synthesis due to their mild reaction conditions and environmental friendliness (Lia al. 2024; Hu et al. 2018). In addition, microbial cell catalysis offers distinct advantages over purified enzyme catalysis, including catalyst recycling and co-factor regeneration (Lin and Tao 2017). There are documented examples of microbial cells that can convert HMF into BHMF, HMFCA, or FDCA (Baptista et al. 2021a; Saikia et al. 2021), but knowledge is still limited (Prasad et al. 2023).

*Saccharomyces cerevisiae*, the most commonly used microorganism in second-generation bioethanol processes, has been extensively studied for its capacity to detoxify HMF and furfural (Cunha et al. 2019, 2020b; Liu 2021). However, the potential of *S. cerevisiae* to produce high-value HMF derivatives has not been thoroughly explored (Baptista et al. 2021b). Notably, under ethanol-producing conditions, the dominant detoxification pathway in *S. cerevisiae* involves the reduction of furans to their respective alcohols. Consequently, reduction processes have been more comprehensively studied than the oxidation of furans (Ishii et al. 2013; Lewis Liu et al. 2008; Nilsson et al. 2005).

Taking all of this into consideration, combining microwave-based biomass processing for HMF production with subsequent whole-cell biocatalysis to produce HMF derivatives represents an integrative approach that has yet to be explored. This study aims to investigate the potential of the yeast *S. cerevisiae* for converting HMF into valuable derivatives. In line with bioeconomy principles and acknowledging the significance of wine residues, these residues were selected for pretreatment using green technologies to produce HMF. The resulting HMF-rich streams were then used to assess the effectiveness of *S. cerevisiae* as a whole-cell biocatalyst. Additionally, the heterologous expression of an HMF oxidase from *Cupriavidus basilensis* HMF14 was evaluated in a selected strain to demonstrate its HMF-oxidizing capability.

## Materials and methods

### Wine byproducts processing for HMF production

Must surplus and grape pomace, kindly provided by the Center of Biofuels and Bioproducts, Agrarian Institute of Castilla and León (Itacyl), were used in this work as substrates to produce HMF. The composition of the must surplus was determined by Hijosa-Valsero et al. (2021) and it was composed of 125 g/L of glucose and 119 g/L of fructose. On the other hand, the composition of grape pomace (expressed as g/100 g of oven-dry weight raw material) was: 7.36 g of glucan. 4.84 g of hemicelluloses, 17.43 g of soluble sugars (composed of 8.62 g of glucose and 8.81 g of fructose), 30.66 g of lignin and 6.64 g of ashes. Both raw materials were processed using a Speedwave 4 microwave digester at temperatures of 195, 225, 250 and 275 ºC for 5 min and 225 ºC for 30 min. For grape pomace, liquid-to-solid ratios (LSR) of 8, 10 and 12 g of water/g of grape pomace were selected and for must, three conditions were evaluated: undiluted and 2/3 and 1/3 dilutions with water to perform microwave treatments. Liquor obtained from microwave treatment of undiluted must was employed as substrate for whole-cell bioconversion of HMF, as described in Sect. 2.3.

### Yeast and bacterial strains, plasmids and genetic modification

The strain employed in this work for plasmid construction, maintenance, and propagation was *Escherichia coli* NZY5α (provided by Nzytech, Portugal). The strains and plasmids of *S. cerevisiae* utilized in this study are detailed in Table 1. The assembly of plasmids was conducted using the In-Fusion HD Cloning Kit from Clontech, USA. The primers utilized for the construction of the plasmid and for confirming integration are listed in the Supplementary material. The expression plasmid pHMFH_cb was constructed based on the plasmid pI23-BGL1-kanMX (Cunha et al. 2020a). The expression cassette included the SED1 promoter, the SAG1 terminator and the coding sequence from the enzyme. The gene encoding HmfH from *C. basilensis* HMF14 (ADE20408.1) was synthesized by NZYTech (Portugal) and was optimized for expression in *S. cerevisiae*. Yeast strains were transformed by the lithium acetate method (Chen et al. 1992) with the constructed plasmid, linearized with BstZ17I. The transformed cells were selected on YPD plates supplemented with G418 (300 mg/L), and the correct integration was verified through PCR from colonies. The newly constructed yeast was labeled ER-cbHMFH.

**Table 1:** *Saccharomyces cerevisiae* strains and plasmids used in this work

	Relevant information	Source
*Saccharomyces cerevisiae* strains		
Ethanol Red (ER)	Commercial yeast for ethanol production	Fermentis, S. I. Lesaffre, Lille ; Lip et al. 2020; Pinheiro et al. 2020
PE-2	Pedra 2 yeast, Brazil ethanol production	Basso et al. 2008
CAT-1	Catanduva 1 yeast, Brazil ethanol production	Basso et al. 2008
CA11	Isolated from the “cachaça” fermentation process of a distillery in Brazil	Schwan et al. 2001
ER-cbHMFH	ER, pI23-HMFH_cb	This work
Plasmids		
pI23-BGL1-kanMX	SED1p–SED1ss–*Aspergillus aculeatus* BGL1–SAG1a–SAG1t, *KanMX* marker, I23 integration site	Cunha et al., 2020
pI23-HMFH_cb	SED1p–*Cupriavidus basilensis* HMFH–SAG1t, *KanMX* marker, I23 integration site	This work

### HMF-derivatives production by whole-cell bioconversion

The *S. cerevisiae* cells were cultivated in yeast peptone dextrose (YPD) medium for either 24–72 h at a temperature of 30 °C with orbital agitation set at 200 rpm, followed by collection through centrifugation at 1000 g for 5 min. Cells underwent a washing procedure with water and were subsequently resuspended in a phosphate buffer (50 mM and at pH 7). The experiment media comprised 50 mM of HMF in a phosphate buffer (50 mM and at pH 7) (named synthetic medium) or HMH-enriched liquor obtained from winery by-products, inoculated with 100 g/L of wet yeast. The experiments of bioconversions were conducted in 6-well microplates placed in an orbital shaker (30 °C and 200 rpm) and utilizing 4 mL of working volume.

### Analytical methods

The bioconversion assay samples underwent analysis for the HMF determination and their derivatives (HMFCA, DFF, FFCA, and FDCA) by HPLC, utilizing an Aminex HPX-87 H column (Bio-Rad) at a temperature of 60 °C, with a 0.01 M H_2_SO_4_ mobile phase and a flow rate of 0.6 mL/min. On the other hand, the selected wavelength for the UV detector was 268 nm, yielding retention times of 18.7 min for FDCA, 22.4 min for HMFCA, 25.6 min for FFCA, 32.7 min for HMF, and 40.5 min for DFF. Samples containing BHMF were analyzed using reverse-phase UHPLC with a Zorbax Eclipse XDB-C18 column (4.6 mm × 250 mm, 5 μm). The analysis was conducted at 25 °C, employing a mixture of acetonitrile/0.4% (NH_4_)_2_SO_4_ (10:90, v/v) at pH 3.5, and a flow rate of 0.6 mL/min. The retention time of BHMF, with a maximum absorption wavelength of 223 nm, was recorded at 11.3 min. The yield (%) is characterized as the ratio of the quantity of a particular HMF derivative to the maximum theoretical quantity of that specific HMF derivative that can be obtained from the initial quantity of HMF. The conversion of HMF (%) was determined by calculating the ratio of the converted HMF to the initial quantity of HMF. The evaporation rate associated with the use of microplates for bioconversion assays was experimentally determined to be 0.168 mL/day. This rate was taken into account when determining the concentrations of HMF and its derivatives. Grape pomace was analyzed for polysaccharides and soluble sugars following standard NREL procedures (Sluiter et al. 2008).

## Results and discussion

### HMF production from wine byproducts

**Fig. 2:**
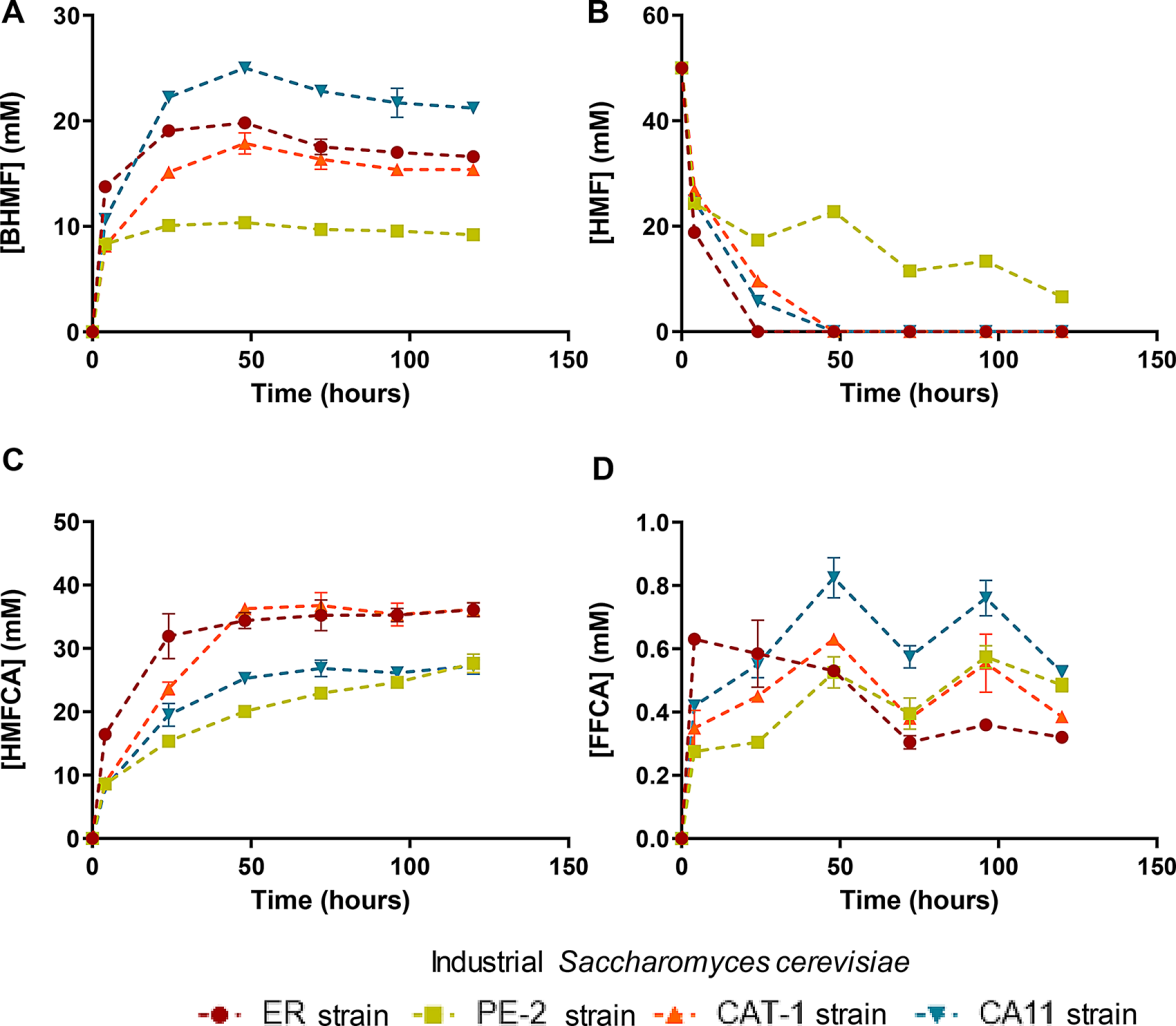
Bioconversion profile of HMF with *Saccharomyces cerevisiae* wild-type strains: ER – Ethanol Red, commercial yeast for ethanol production; PE-2- Pedra 2 yeast, Brazil ethanol production; CAT-1 - Catanduva 1 yeast, Brazil ethanol production; CA11- Isolated from the “cachaça” fermentation process of a distillery in Brazil. Data represents mean ± standard deviation of two biological replicates

Wine residues, must surplus and grape pomace, were selected for their high sugar content (as hexoses and/or as polysaccharides) to produce HMF from a renewable biomass. Initially, preliminary experiments were conducted using microwave treatment at 190 ºC for 5 min, with uncatalyzed water as the reaction medium, to assess the potential of these biomasses for HMF production. As shown in Fig. 2A, the HMF concentration reached 30 mM from must surplus and remained below 10 mM for grape pomace. Due to the insufficient pretreatment severity, the temperature was subsequently raised to a range of 225 to 275 ºC (Fig. 2B).

Additionally, due to its higher hexose concentration compared to the sugars in grape pomace, must was found to be more suitable for HMF production, as shown in Fig. 2B. Microwave treatment of grape pomace yielded a liquor with an HMF concentration of less than 15 mM at a liquid-to-solid ratio of 8 g/g. In contrast, undiluted must produced up to 70 mM of HMF at 275 ºC for 5 min. Consequently, undiluted must was selected as the most suitable substrate for further HMF and HMF-derivative production using whole-cell *S. cerevisiae* biocatalyst. Although microwave treatment can generate HMF from glucose and fructose, the HMF yield from undiluted must was relatively low (< 20%). To improve this, an additional microwave treatment was performed at 225 ºC, extending the reaction time to 30 min. This adjustment led to a significant increase, yielding 218 mM of HMF, thereby increasing the HMF concentration in the liquor by 10-fold.

### Evaluation of *Saccharomyces cerevisiae* strains for HMF bioconversion

As described in a previous study, strains of *S. cerevisiae* from various backgrounds exhibited unique characteristics, including enhanced robustness and varying abilities for the detoxification of furan compounds (considered inhibitors of yeast growth) such as furfural and HMF (Pereira et al. 2014). Based on this, the following yeast strains: (i) the thermotolerant yeast strain Ethanol Red (ER) (Lip et al. 2020; Pinheiro et al. 2020) developed for the industrial ethanol sector, (ii) the Brazilian first-generation bioethanol strains Pedra 2 (PE-2) (Secches et al. 2022; Romaní et al. 2016) and (iii) Catanduva 1 (CAT-1) (Secches et al. 2022; Pereira et al. 2010), along with (iv) CA11, isolated from a “cachaça” distillery (Costa et al. 2017), were assessed for their ability to convert 50 mM of synthetic HMF medium.

**Fig. 3:**
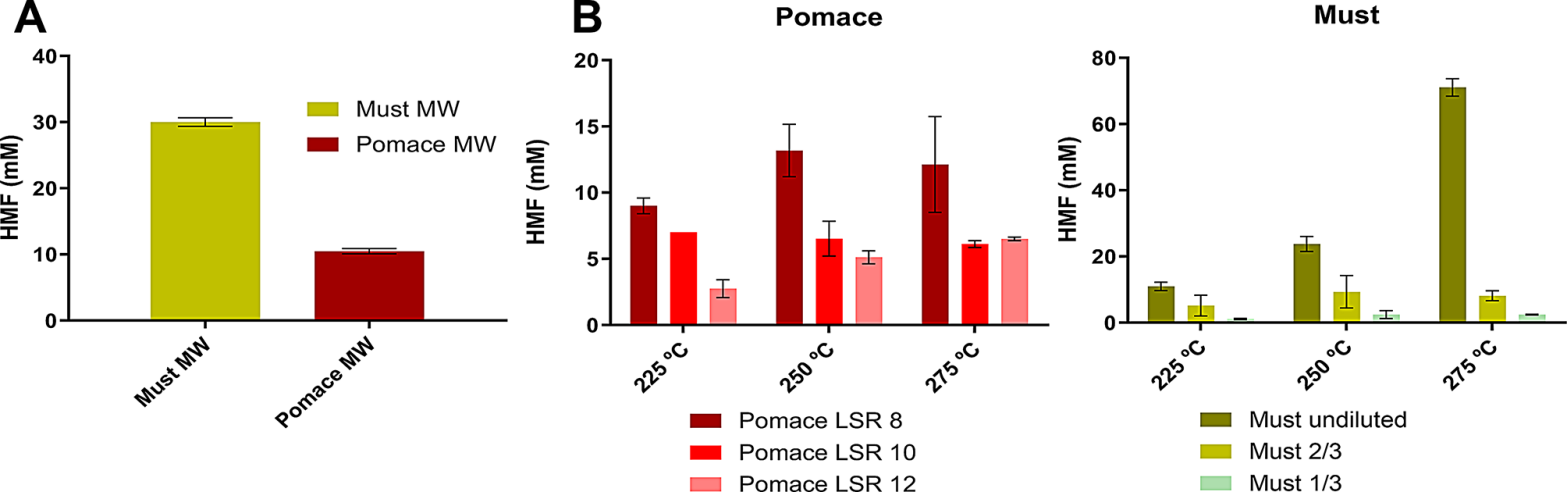
HMF production from grape must and pomace after microwave treatment (MW): **(A)** at 190ºC for 5 min and **(B)** at 225–275 ºC for 5 min at different LSR: Liquid-to-solid ratio

HMF consumption and the main HMF derivatives obtained from bioconversion assays using these industrial strains are shown in Fig. 3A and D. It is noteworthy that BHMF concentration rises until 48 h of conversion, followed by a slight decline until 120 h (Fig. 3A). The primary mechanism of yeast to detoxify HMF is the production of BHMF during the early phases of the bioconversion assay, with a gradual conversion into HMFCA. This behaviour was also previously reported for the metabolism of furfural by *S. cerevisiae* (Taherzadeh et al. 1999, 2000).

In relation to other oxidized HMF derivatives, the yeast strains yielded minimal quantities of FFCA (≤ 0.8 mM, Fig. 3D) and FDCA (≤ 0.5 mM, data not shown). These results indicate that the oxidation of HMF by *S. cerevisiae* strains occurs exclusively via the HMFCA intermediate (Route A shown in Fig. 1) with no DFF being produced during the bioconversion experiment. Considering the high accumulation of HMFCA (Fig. 3C) and the low concentrations of FFCA achieved (as seen in Fig. 3D), it is reasonable to conclude that these yeast strains lack the capacity to oxidize the hydroxymethyl group of HMF and can only marginally oxidize that group in HMFCA. This fact can be supported by the hypothesis that the oxidation of furfural in yeast is mediated by aldehyde dehydrogenase (Sárvári Horváth et al. 2003), a process that could similarly occur during the oxidation of HMF. It was reported that the overexpression of aldehyde dehydrogenase 6 in *S. cerevisiae* allowed the direct oxidation of furfural and HMF to their corresponding acids by utilizing NADP + and regenerating NADPH (Park et al. 2011).

The yeast strain *S. cerevisiae* Ethanol Red demonstrated the fastest bioconversion of HMF (Fig. 3B) into HMFCA (Fig. 3C) compared to the other three strains. However, there remains significant potential for optimizing the process conditions. This could include adjustments to pH and temperature, as well as the addition of neutralizers and/or co-substrates, since HMFCA production is still far from the theoretical maximum. To the best of the author´s knowledge, no studies have fully clarified the potential and required conditions for the complete oxidation of HMF by the yeast *S. cerevisiae*. Overall, the data presented in Fig. 3 provide the first evidence of *S. cerevisiae*’s ability to accumulate HMFCA from high HMF concentrations (50 mM), underscoring its potential as a biocatalyst for HMFCA production and/or as a platform for synthesizing additional oxidized HMF derivatives. Among the strains tested, Ethanol Red proved to be the most efficient in detoxifying such a high concentration of HMF.

### HMF-derivatives production from HMF-enriched must

The HMF enriched liquor obtained from the microwave treatment of undiluted must (225 ºC for 5 min) was used as a renewable substrate for biocatalysis conversion into HMF-derivatives, using the following catalysts: (i) *S. cerevisiae* Ethanol Red (selected based on previous results) and (ii) *S. cerevisiae* CEN.PK113-7D (a laboratory strain used for comparison). Evaluation of the must hydrolysate (225 ºC for 5 min) using the *S. cerevisiae* Ethanol Red strain showed that HMF was nearly depleted in less than 2 h (HMF decreased from 13.33 mM of HMF to 0.16 mM (data not shown). In contrast, CENPK113-7D required 4 h to achieve equivalent detoxification, reducing HMF from 13.6 mM to 0.15 mM.

**Fig. 4:**
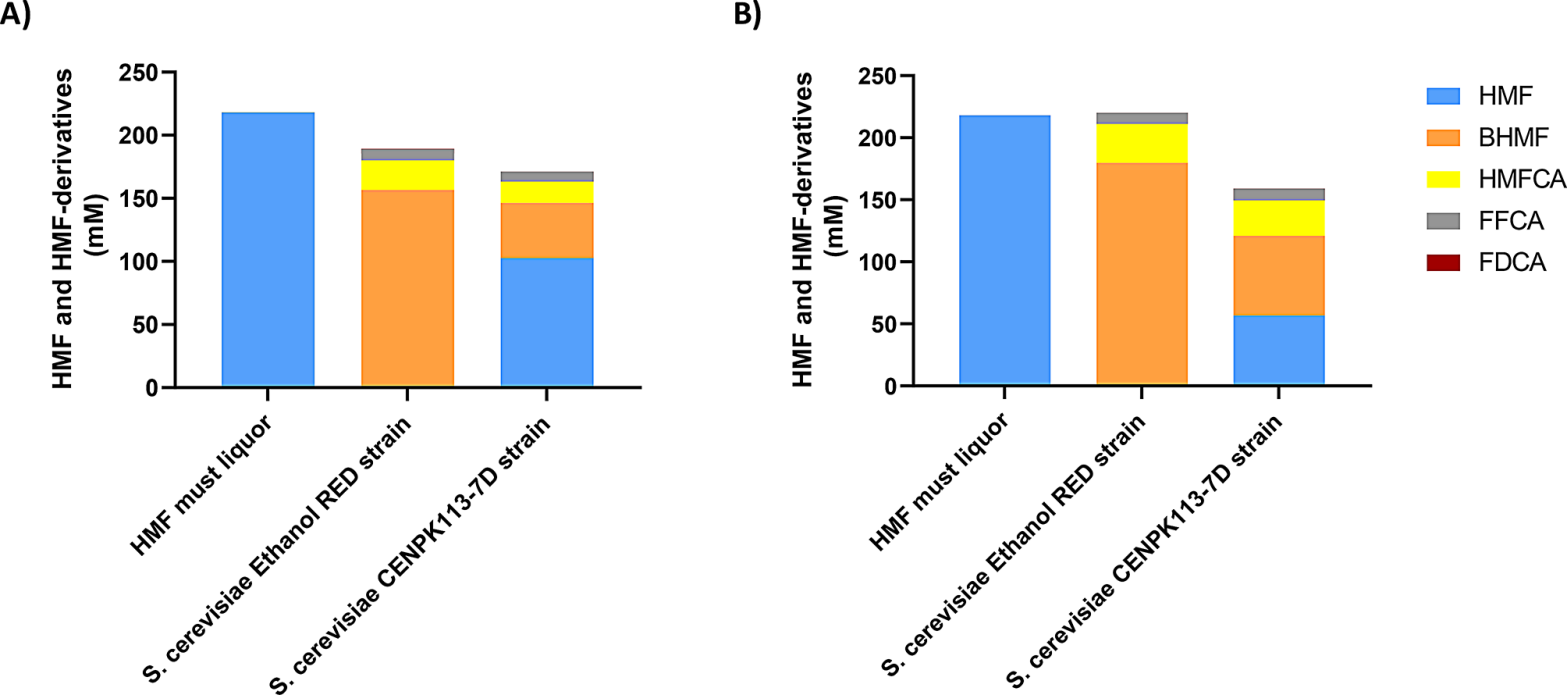
Bioconversion of HMF from microwave treated must (218 mM) into HMF-derivatives: **(A)** at 12 h of whole-cell biocatalysis and **(B)** at 48 h of whole-cell biocatalysis with two selected *Saccharomyces cerevisiae* strains, Ethanol Red and CENPK113-7D. HMF must liquour refers to the control where no yeast strain was added

When using an HMF-rich stream (218 mM) obtained from must surplus treated with environmentally friendly technologies such as microwaves (225 ºC for 30 min), more significant differences are observed between the laboratory strain CENPK113-7D and the industrial strain Ethanol Red (Fig. 4). Notably, the Ethanol Red strain could completely detoxify 218 mM of HMF in just 12 h, whereas the laboratory strain CENPK113-7D, detoxified 170 mM in 48 h. In both strains, the main product is BHMF, but Ethanol Red produces approximately three times more of this compound (179 mM). The second product, HMFCA, is produced at approximately 30 mM by both strains, while the third product, FFCA, is generated in smaller quantities (~ 10 mM). Under high HMF concentrations, the Ethanol Red strain demonstrates superior performance and was chosen as the host chassis for genetic modification to expand the product portfolio to include the key bio-based platform chemical FDCA, which was hardly detected in the current setup.

### Expression of HMF14 (CbHMFH) enzyme to improve FDCA production

**Fig. 5:**
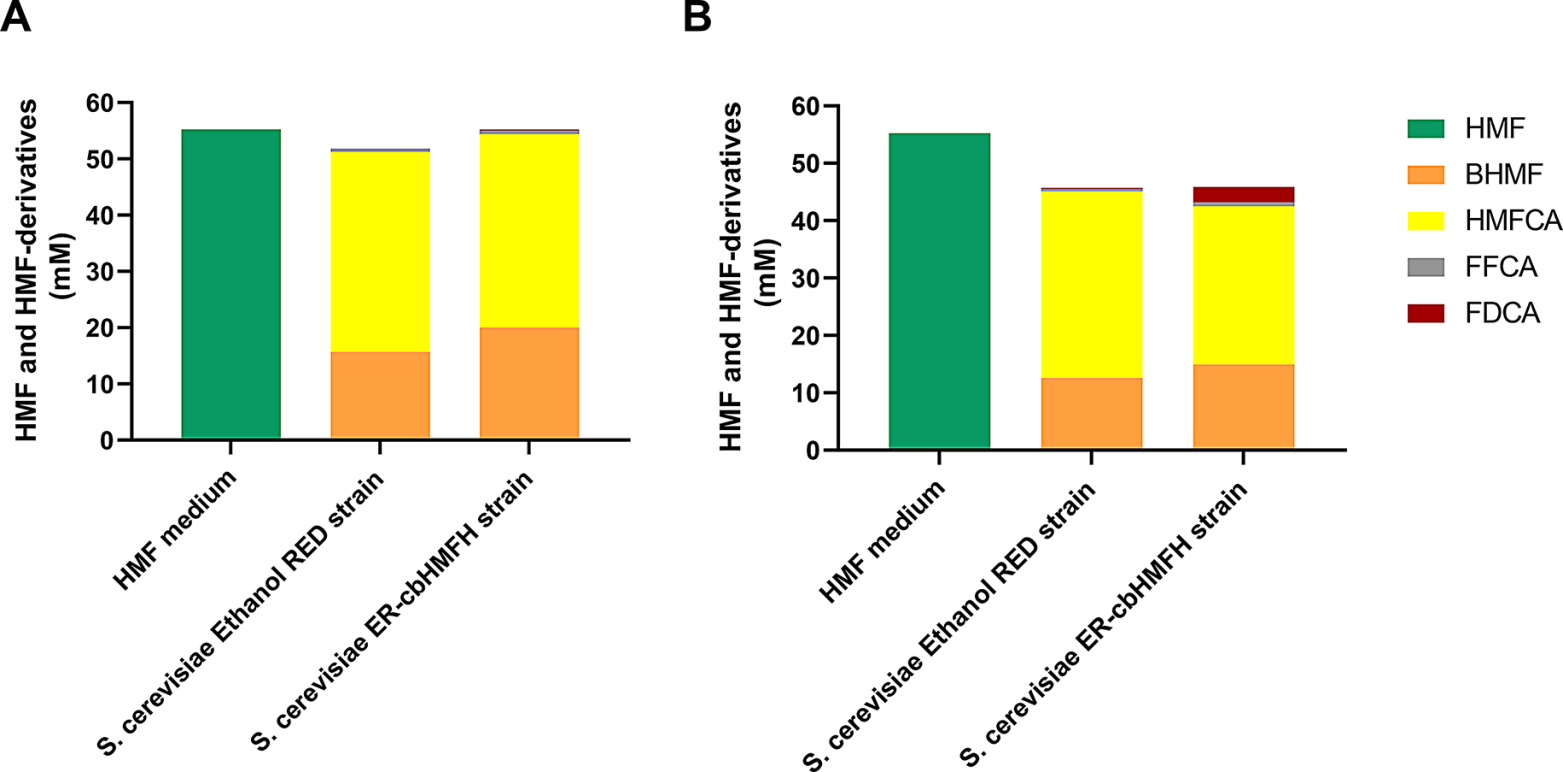
HMF and HMF-derivatives production from HMF enriched medium (55 mM) with *Saccharomyces cerevisiae* Ethanol Red and engineered strain expressing the heterologous HMF oxidoreductase from *Cupriavidus basilensis HMF14* (ER-cbHMFH) **(A)** at 24 h of bioconversion and **(B)** at 120 h of bioconversion. HMF medium refers to the control where no yeast strain was added

In light of these findings that underscore Ethanol Red as a potential biocatalyst for HMF oxidation, this strain was modified to express a heterologous enzyme aimed at FDCA production. FDCA is recognised as one of the 12 top chemicals derived from biomass due to its industrial relevance. The enzyme chosen, HMF/Furfural oxidoreductase from *Cupriavidus basilensis* HMF14 (CbHMFH) is a FAD-dependent oxidoreductase that utilizes oxygen as a co-substrate in oxidation reactions, resulting in the production of H_2_O_2_ (Koopman et al. 2010b; Dijkman and Fraaije 2014). The selection of CbHMFH was based on prior studies demonstrating its successful heterologous expression in the bacteria *Pseudomonas putida* (Koopman et al. 2010a) and *Raoultella ornithinolytica* BF60 (Yuan et al. 2018), where it enabled the conversion of HMF to FDCA under aerobic conditions. Thus, CbHMFH was a logical candidate to test in the aerobic host *S. cerevisiae*. This study marks the first time this enzyme has been expressed in this yeast. The engineered strain, ER-cbHMFH, and the Ethanol Red wild type (as the control) were employed in a bioconversion assay to catalyze the transformation of 50 mM of HMF (Fig. 5). As anticipated, employing ER-cbHMFH led to accelerated oxidation of HMF (Koopman et al. 2010b), resulting in quicker production of HMFCA. However, it also led to an unexpected increase in the production and accumulation of BHMF (Fig. 5). Despite these challenges, ER-cbHMFH significantly enhanced FDCA production, achieving quantities 15 times greater than those produced by the control strain ER. The final pH of the bioconversion medium was lower for the engineered strain, ER-cbHMFH (pH 3.65), compared to the wild-type strain (pH 4.35). This pH drop is likely due to FDCA accumulation, which acidifies the medium and could hinder the extent of the bioconversion. pH-related challenges in whole-cell biocatalysis are well-documented, as metabolic product accumulation can lead to acidification. Strategies such as the addition of pH neutralizers, like calcium carbonate, could mitigate this issue and enhance conversion efficiency (Sheng et al. 2020; Xu et al. 2020). Literature collects several strategies for the genetic engineering of *S. cerevisiae* to obtain HMF derivatives. For instance, the heterologous expression of alcohol dehydrogenases from *Meyerozyma guilliermondii* in *S. cerevisiae* for the production of BHMF from HMF was recently investigated by Xia and co-workers (2020). The recombinant *S. cerevisiae* strain exhibited greater productivity (15 mM/h over 23 h in a fed-batch strategy) compared to the bioconversion using whole cells of *M. guilliermondii* (Li et al. 2017). On the other hand, the only report utilizing *S. cerevisiae* as a biocatalyst for producing oxidized HMF derivatives is a patent evaluating several fungal species as hosts for FDCA production (De Bont 2018). In this study, a laboratory strain of *S. cerevisiae* was engineered to express HMF/Furfural oxidoreductase and HMF/FFCA dehydrogenase derived from *C. basilensis* HMF14, or alternatively, to produce alcohol dehydrogenase and aldehyde dehydrogenase sourced from *Penicillium brasilianum*. These strategies resulted in the generation of 0.21 and 3.02 mM FDCA from approximately 4 mM of HMF, respectively, which are lower substrate concentrations and FDCA titers compared to the results achieved in this study.

## Conclusions

This work establishes *S. cerevisiae* as a biocatalyst for converting HMF into its oxidized derivatives, highlighting notable variations among strains. Green technologies were employed to obtain HMF-enriched liquor from wine residues, which served as the substrate. The selected Ethanol Red yeast strain was engineered to express a heterologous HMF/Furfural oxidoreductase to enhance HMF oxidation. While the engineered strain accumulated BHMF and HMFCA at levels comparable to the wild-type strain, it achieved a 15-fold increase in FDCA titers. This work represents the first demonstration of *S. cerevisiae* producing HMF derivatives at relevant titers using wine residues as a feedstock.

E-supplementary data of this word can be found in online version of the paper.

## Electronic supplementary material

Below is the link to the electronic supplementary material.

Supplementary Material 1

Supplementary Material 2

## Data Availability

Not applicable.

## Abbreviations

BHMF: 2,5-bis(hydroxymethyl)furan
DFF: 2,5-diformylfuran
HMF: 5-hydroxymethylfurfural
HMFCA: 5-hydroxymethyl‐furan‐2‐carboxylic acid
FDCA: 2,5-furandicarboxylic acid
FFCA: 5-formyl 2‐furancarboxylic acid
LSR: Liquid-to-solid ratios
PET: Polyethylene terephthalate
