# Supplementary figures and images for: Production of HMF-derivatives from wine residues using Saccharomyces cerevisiae as whole-cell biocatalyst
Source: Bioresour Bioprocess. 2025 Jan 31;12(1):8. doi: 10.1186/s40643-025-00840-5 (PMC11785874; doi:10.1186/s40643-025-00840-5)

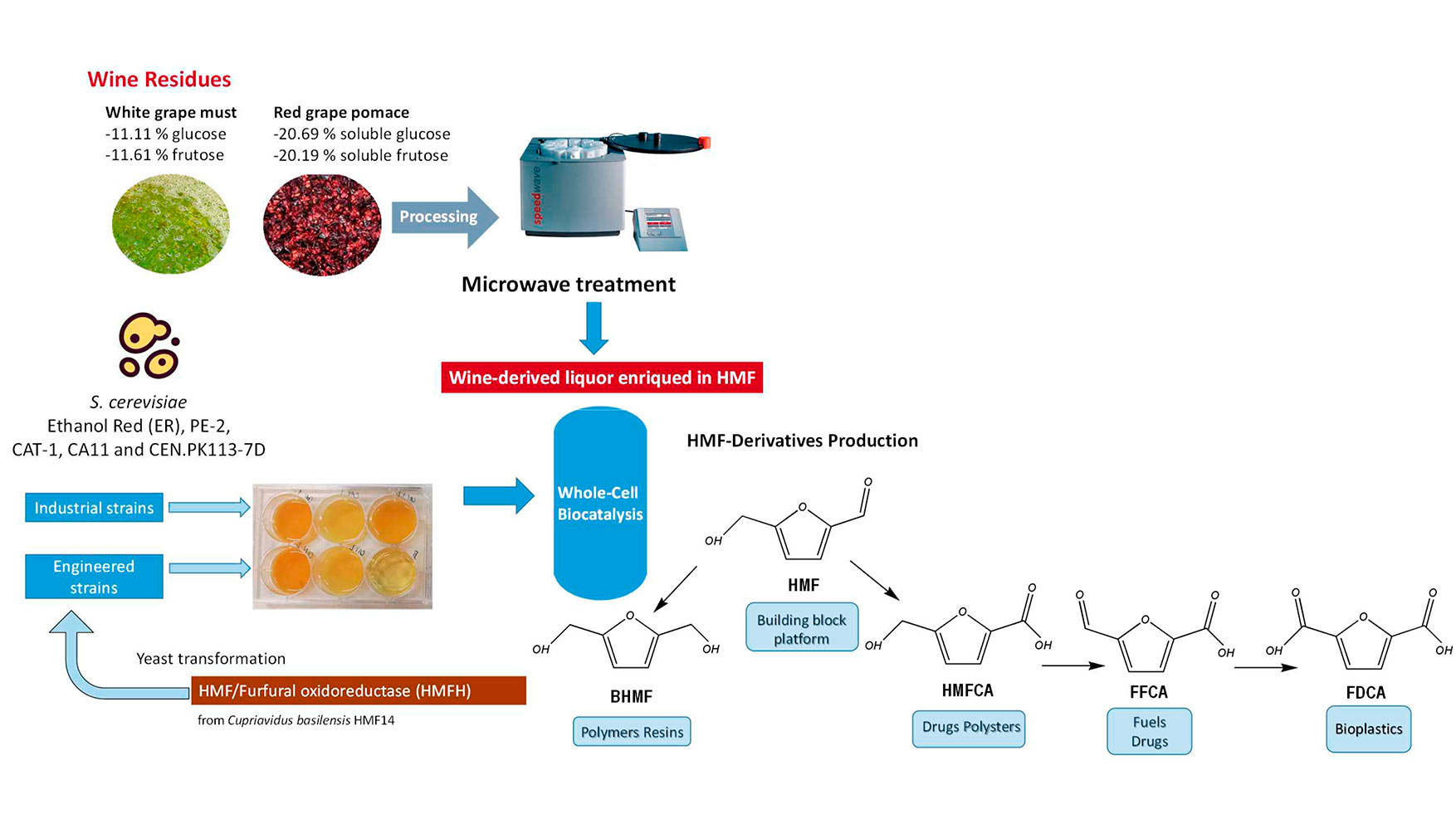

Supplement: Supplementary file 1 — Supplementary Material 1 [file 40643_2025_840_MOESM1_ESM.jpg]
