# Supplementary material for: Production of HMF-derivatives from wine residues using Saccharomyces cerevisiae as whole-cell biocatalyst
Source: Bioresour Bioprocess. 2025 Jan 31;12(1):8. doi: 10.1186/s40643-025-00840-5 (PMC11785874; doi:10.1186/s40643-025-00840-5)
Supplement: Supplementary file 2 — Supplementary Material 2 [file 40643_2025_840_MOESM2_ESM.docx]

**Supplementary Data**

**Production of HMF-Derivatives from Wine Residues Using *Saccharomyces cerevisiae* as whole-cell biocatalyst**

**Table S.1.** List of primers used for subcloning steps in this work. Lower case sequences indicate addition of homologous regions for plasmid assembling

| **Primers** | **Sequence (5’->3’)** | **Aim** |
| --- | --- | --- |
| PI23_simple_fw | AACGGGTACTGTACAGTTAG | Amplification of pI23-BGL1-kanMX for construction of plasmids for intracellular production of *Cb*HMFH |
| PI23_simple_rv | CATCTTAATAGAGCGAACGTA |  |
| pFDCA_cup_PI23_fw | cgctctattaagATGGACACTCCGAGAGAAAG | Amplification of the coding sequence of HMF/Furfural oxidoreductase from *Cupriavidus basilensis* HMF14 (*Cb*HMFH) for intracellular expression |
| pFDCA_cup_PI23_rv | tgtacagtacccgttTTACGATGGGTGCGCGAC |  |
| I23_FW | ATAATGAGTTCCGAGTCTGTTGGTG | Confirm integration in intergenic region I23 |
| I23_RV | CGAGATAAGGCATGGGGTTCTG |  |
